# Criminal convictions in males and females diagnosed with attention deficit hyperactivity disorder: A Swedish national registry study

**DOI:** 10.1002/jcv2.12217

**Published:** 2024-01-20

**Authors:** Anna‐Karin Ångström, Anneli Andersson, Miguel Garcia‐Argibay, Zheng Chang, Paul Lichtenstein, Brian M. D’Onofrio, Catherine Tuvblad, Laura Ghirardi, Henrik Larsson

**Affiliations:** ^1^ School of Medical Sciences Örebro University Örebro Sweden; ^2^ School of Psychology, Law and Social Work Örebro University Örebro Sweden; ^3^ Department of Medical Epidemiology and Biostatistics Karolinska Institutet Solna Sweden; ^4^ Department of Psychological and Brain Sciences Indiana University Bloomington Indiana USA; ^5^ Department of Psychology University of Southern California Los Angeles California USA; ^6^ MediNeos Observational Research ‐ IQVIA, Data Management & Statistics Modena Italy

**Keywords:** ADHD, non‐violent crime, violent crime

## Abstract

**Background:**

Individuals with Attention‐Deficit/Hyperactivity Disorder (ADHD) face an elevated risk of criminal convictions compared to those without ADHD. However, understanding this link involves considering sex differences, coexisting psychiatric conditions, and unmeasured familial factors. This study aimed to explore the connection between ADHD and criminal convictions (both violent and non‐violent) in males and females, while also assessing the impact of comorbid psychiatric disorders and familial factors.

**Methods:**

Using Swedish national registers, we identified individuals born between 1986 and 1997 (635,391 males and 600,548 females). ADHD was defined through clinical diagnosis and prescribed medications, while criminal convictions were determined based on Swedish lower court records. Unmeasured familial factors were accounted for using a sibling design approach.

**Results:**

Findings revealed that individuals with ADHD had a notably higher absolute and relative risk of both violent and non‐violent criminal convictions compared to those without ADHD. While criminal convictions were more frequent among males with ADHD, females with ADHD exhibited higher relative risks (HR violent 10.50, non‐violent 4.04) than their male counterparts (HR violent 6.03, non‐violent 3.57). Additionally, lower socioeconomic status (SES) in individuals with ADHD was associated with increased relative risks for criminal convictions compared to individuals with ADHD who had higher SES. Adjusting for childhood and internalizing psychiatric disorders partially attenuated these associations, while substance use disorders (SUD) substantially attenuated them. SUD also contributed to an elevated absolute risk of criminal convictions in both male and female individuals with ADHD. Accounting for unmeasured shared familial factors slightly reduced the estimates, but the association between ADHD and criminal convictions persisted.

**Conclusion:**

In conclusion, ADHD remains a potent independent risk factor for criminal convictions, with varying effects based on gender. This underscores the importance of tailored crime prevention strategies and early interventions for individuals with ADHD, especially when comorbid SUD is present.

## Graphical abstract

Individuals diagnosed with ADHD are at an elevated risk of criminal convictions, and this risk varies by gender, with females with ADHD exhibiting higher relative risks than males. Notably, the presence of comorbid substance use disorders significantly magnifies this risk, and individuals with ADHD and lower socioeconomic status (SES) face a heightened risk compared to their counterparts with higher SES. Importantly, even after adjusting for familial factors, the association between ADHD and criminal convictions remains, emphasizing the need for personalized interventions, particularly for those with comorbid substance use disorders.

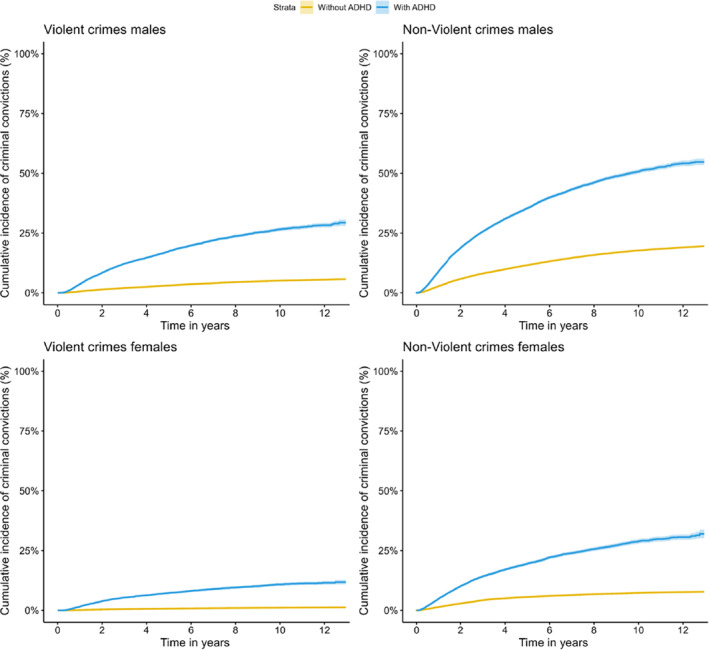

**Key points**

- Previous research has established that ADHD is associated with higher rates of criminality.
- Even after adjusting for SES, comorbid psychiatric disorders and unmeasured familial factors, ADHD is an important risk factor for violent and non‐violent criminal convictions.
- Females with ADHD showed higher relative risk for criminal convictions and males with ADHD higher absolute risk for criminal convictions.
- Substance use disorder attenuated the association between ADHD and criminal convictions substantially. SUD also increased the absolute risk of being convicted of a violent or non‐violent crime in individuals with ADHD.
- Considering the elevated risk of criminal convictions, individuals with ADHD and especially those with comorbid SUD should be target for prevention and treatment.

## INTRODUCTION

Attention Deficit Hyperactivity Disorder (ADHD) is a neurodevelopmental condition characterized by symptoms of inattention, hyperactivity, and impulsivity (American Psychiatric Association, 2013). Worldwide, ADHD affects approximately 2%–7% of children (Sayal et al., 2018) with persisting ADHD related problems into adulthood for 50%–90% of cases (Faraone et al., 2006; Lara et al., 2009; Sibley et al., 2022). Previous research suggests that ADHD frequently co‐occurs with other psychiatric disorders, including substance use disorder, autism spectrum disorders, conduct disorder, depression, and anxiety (Andersson et al., 2020; Demontis et al., 2019; Kessler et al., 2006; Sobanski et al., 2007). The association between ADHD and criminality is well‐established through studies based on self‐reports (De Sanctis et al., 2014; Koisaari et al., 2015; Whiting et al., 2021). However, self‐reports in community samples suffer from important limitations, including recall bias and non‐representative sampling due to low participation rates among the most severe cases. Fewer studies have used population‐based samples with information on recorded criminal convictions, which is collected prospectively and independently from the information about ADHD. This is an important limitation given that criminal convictions are a concrete, public health relevant outcome measure that could be used to better understand the overall burden of ADHD.

A meta‐analysis and systematic review including 11 studies found that childhood ADHD (*n* = 15,442) was associated with a three‐fold increased risk for criminal convictions, but the meta‐analysis only included two large‐scale (>1000 individuals with ADHD) studies (Mohr‐Jensen & Steinhausen, 2016). The first study observed that individuals with ADHD had an almost three‐fold increased risk of violent criminal convictions after adjusting for confounders (e.g., parental education, SES, and SUD) (Lundström Forsman, Larsson, 2014). They also reported that unaffected siblings of individuals with ADHD had an increased risk of violent criminal convictions (Lundström et al., 2014), indicating that familial factors (i.e., genetic and environmental factors making siblings similar) influence the association between ADHD and violent criminal convictions. The second study found that both males and females with ADHD showed a 2‐fold increased risk of being convicted of any crime (Silva et al., 2014).

More recently, a large Danish population‐based register study examined the association between childhood ADHD (age range 4–15 years) and criminal convictions. After adjusting for various risk factors (e.g., psychiatric comorbidity, parental education, and SES), ADHD was associated with a 1.6‐fold increased risk of criminal convictions (Mohr‐Jensen et al., 2019). In contrast to the study by Silva et al. (2014), Mohr‐Jensen et al., 2019 found that female sex was associated with increased risk of criminal conviction in individuals with ADHD (Mohr‐Jensen et al., 2019). However, as only 15% of those with a diagnosis of ADHD were females (Mohr‐Jensen et al., 2019), these results need to be replicated using a larger sample to yield more precise estimates.

Mohr‐Jensen et al., 2019 also reported that the presence of co‐occurring externalizing psychiatric disorders was associated with almost three times higher risk of criminal convictions compared to those with ADHD only. The risk of criminal convictions was particularly pronounced in individuals with ADHD and co‐occurring SUD or conduct disorders (Mohr‐Jensen et al., 2019). More research is needed to firmly establish the specific role of psychiatric comorbidities, including SUD. Such knowledge is needed to identify crime prevention strategies, and early intervention targets for individuals with a diagnosis of ADHD.

Previous research has found that genetic factors are an important part of the etiology of ADHD with heritability estimates between 70%—80% (Faraone & Larsson, 2019), which may impart explain the observed association between ADHD and criminal convictions. Genetically‐sensitive study designs (e.g., sibling comparison) are therefore needed to explore the extent to which ADHD influences the risk of criminal convictions via individual‐specific factors (i.e., factors not shared within families) or shared genetic/familial factors.

In this register‐based cohort study, we aimed to fill these knowledge gaps by investigating the prospective associations between ADHD and the risk of violent and non‐violent criminal convictions. We examined potential sex differences and the extent to which any observed associations could be explained by co‐occurring psychiatric disorders. Finally, using a sibling comparison approach, we explored the role of unmeasured familial factors.

## METHOD

### Study population

The study had ethical approval from the Regional Ethical Review Board in Stockholm, Sweden (Dnr 2013/862‐31/5). We used a linkage of Swedish national registers to identify all individuals born between 1986 and 1997 (*N* = 1,646,645). We excluded individuals that had died (*n* = 9566; 0.8% of the target population) or emigrated (*n* = 77,943%; 6.3% of the target population) before their 15th birthday (which is the minimum age of criminal responsibility in Sweden). An additional 323,197 individuals (26.2% of the target population) were excluded as they had immigrated to Sweden. These individuals were excluded because information about their psychiatric disease history (e.g., ADHD) or information about criminal conviction may be incomplete and induce information bias. Descriptive information on exposure, covariates, and outcomes on those excluded is presented in Supplementary Table 1. The final cohort consisted of a total of *n* = 635,391 males and *n* = 600,548 females, resulting in a total of 1,235,939 individuals.

### Exposure

The Swedish National Patient Register (NPR; Ludvigsson et al., 2011) and the Prescribed Drug Register (PDR; Wettermark et al., 2007) were used to define ADHD. Based on information from these two registers, ADHD was defined as either having received a clinical diagnosis according to the International Classification of Disease (ICD‐9 314 and ICD‐10 F90) or having received a prescription of medications used to treat ADHD according to Anatomical Therapeutic Chemical (ATC; amphetamine (N06BA01), dexamphetamine (N06BA02), methylphenidate (N06BA04), atomoxetine (N06BA09) or lisdexamphetamine (NO6BA12)). Previous research has indicated high specificity for this register‐based ADHD definition in Sweden (Larsson et al., 2013) and found that patterns of etiological influences remain similar whether people with ADHD are identified through diagnoses or prescriptions (Larsson et al., 2014). This definition resulted in *n* = 27,103 (4.3%) males and *n* = 15,960 (2.7%) females being diagnosed with ADHD, a total *n* = 43,063 (3.5% of the included population, 37% females).

### Outcomes

Criminal convictions in Sweden between 2001 and 2013 were identified using the National Crime Register. The convictions were divided into violent convictions and non‐violent convictions. In line with previous research (Frisell et al., 2011) violent convictions were defined as any conviction of homicide, manslaughter, assault, robbery, threats and violence against an officer, unlawful threats, unlawful coercion, kidnapping, illegal confinement, arson, a gross violation of a person's integrity, recurrent intimate partner violence directed toward a woman, intimidation or sexual offenses (including rape, indecent assault, indecent exposure or child molestation, but excluding prostitution, hiring of prostitutes or possession of child pornography), but also additional convictions such as causing another's death, riot, interference in a judicial matter and other related crimes. Non‐violent convictions were defined as all convictions not included among violent convictions, as defined previously (Frisell et al., 2011; Kuja‐Halkola et al., 2012).

### Covariates

Information about sex, year of birth, and family relationships (i.e., biological parents) was retrieved from the Total Population Register (TPR). Socioeconomic status (SES) was assessed using information about the parents' highest level of education when the child turned 15 years old and was retrieved from the Longitudinal Integrated Database for Health Insurance and Labor Market Studies (LISA) (Ludvigsson et al., 2019). This variable was categorized as: <9 years, 9 years, 10–11 years, 12 years, 13–14 years, 15 years, and >15 years (Ludvigsson et al., 2019) and further categorized into three levels of socioeconomic status were ≤9 years of education became Level 1, >10–14< years Level 2, and ≥15 years of education Level 3. The Swedish Multi‐Generation Register (Ekbom, 2011) was used to link the index persons to their full siblings. The NPR was used to define the presence of comorbid psychiatric disorders, and these were divided into subgroups depending on their nature. Autism spectrum disorders (ASD), intellectual disability (ID), conduct disorder (CD), and oppositional defiant disorder (ODD) were categorized as childhood psychiatric disorders (Wills, 2014). Depression and anxiety disorders were categorized as internalizing psychiatric disorders and considered to be mediators. Substance use disorder (SUD) was also considered as a mediator but was not divided into any subgroup due to the unique association between ADHD and SUD (Frodl, 2010; Martinez‐Raga et al., 2013). For transparent and complete reporting of covariates (Larsson, 2022) all ICD‐codes used to identify the presence of comorbidity are presented in Supplementary Table 3.

## STATISTICAL ANALYSES

To illustrate the cumulative incidence of criminal convictions during the follow‐up, we used one minus the Kaplan‐Meier estimate of the survival function. Individuals were followed from age 15 until criminal conviction, death, emigration, or end of follow‐up (December 2013), whichever occurred first. ADHD was modeled as a time‐varying exposure; that is, individuals were assigned to the unexposed group before the diagnosis of ADHD and were assigned to the exposed group from the first diagnosis of ADHD or ADHD medication prescription to the end of follow‐up. All covariates (for example ASD and SUD) were modeled as time fixed, meaning that the diagnoses could be received at any time during the follow up period. Criminal convictions were assessed using the first conviction of violent and non‐violent crimes after the individuals turned 15 years. To explore the association between ADHD and criminal convictions, we used Cox proportional hazard models with 95% Cis to estimate HRs, with time from the individual's 15^th^ birthday as the underlying timescale. First, we estimated the association of ADHD with violent and non‐violent criminal convictions in males and females diagnosed with ADHD versus those without ADHD. Second, we adjusted for the year of birth and SES. Third, we adjusted for year of birth, SES, and childhood psychiatric disorders (ASD, ID, CD, and ODD). In the next step, we adjusted for year of birth, SES, and internalizing disorders (depression and anxiety). Finally, we adjusted for birth year, SES and SUD. We illustrated the cumulative incidence of criminal convictions during the follow‐up in individuals with ADHD only compared to those with ADHD and comorbid SUD using one minus the Kaplan‐Meier estimate of the survival function. We further used an interaction term to examine if the risk of a criminal conviction in males and females and within individuals with different levels of SES with ADHD significantly differed.

To estimate the association between ADHD and violent and non‐violent criminal convictions in individuals diagnosed with ADHD after adjustment for unmeasured familial factors, we used stratified Cox proportional hazard models, conditioning on full sibling pairs (Allison, 2009). If the association between ADHD and criminal convictions is influenced by shared familial factors, the comparison of differentially exposed sibling pairs will result in attenuated estimates. Due to the limited sample of females with ADHD, this analysis could not be conducted separately for males and females. Data management and descriptive analyses were performed with SAS software version 9.4 (SAS Institute Inc., Cary, NC) and R 3.6.1 (R Development Core Team, 2020).

### Sensitivity analyses

#### ADHD defined at any time

ADHD is a condition that typically has its onset in childhood, but some receive their ADHD diagnosis later in life (Agnew‐Blais et al., 2016; Caye et al., 2016; Kessler et al., 2006; Moffit, 2016; Riglin et al., 2016), see Supplementary Table 2 for information regarding mean age of diagnosis in the included sample. Swedish registers started to include information about outpatient healthcare in 2001, which could also contribute to later ADHD diagnoses. This may introduce bias due to exposure misclassification. For example, some individuals assigned to the unexposed group until diagnosis might have undiagnosed ADHD and thereby should be in the exposed group (i.e. false negatives). This potential misclassification may attenuate associations toward the null. To account for this, we conducted a set of sensitivity analyses by treating exposure to ADHD as a non‐time‐bound variable, that is, participants receiving their ADHD diagnosis after age 15 were considered as exposed from the start of the follow‐up at age 15.

#### Pharmacological treatment of ADHD

Previous research has shown that treatment with ADHD medication may decrease the risk of criminal convictions (Lichtenstein et al., 2012). We therefore excluded individuals receiving pharmacological treatment for ADHD prior to any criminal conviction and compared the effect sizes with the results from the main analyses.

## RESULTS

The final cohort consisted of 1,235,939 individuals, 635,391 males (51.4%) and 600,548 females (48.6%), the average (mean), full sample follow‐up time for violent criminal convictions was 6.95 (Standard Deviation; SD = 3.36) years and 6.54 (SD = 3.47) years for non‐violent criminal convictions. A total of 27,103 (4.3%) males and 15,960 (2.7%) females were defined as having ADHD. The average follow‐up time among individuals with ADHD were 5.56 years for violent criminal convictions and 4.85 years for non‐violent criminal convictions. Among males and females with a violent or non‐violent criminal conviction, individuals with ADHD were more likely to also be diagnosed with another psychiatric disorder, compared to males and females without ADHD, Table 1.

**TABLE 1:** Prevalence of ADHD, psychiatric comorbidities, and criminal convictions in the study population by sex.

	Full sample (*n* = 1, 235,939)		*p*‐value, effect size	Males (*n* = 635,391)		*p*‐value, effect size	Females (*n* = 600,548)		*p*‐value, effect size
	ADHD (*n* = 43,063)	Non‐ADHD (*n* = 1, 192,876)		ADHD (*n* = 27,103)	Non‐ADHD (*n* = 608,288)		ADHD (*n* = 15,960)	Non‐ADHD (*n* = 548,588)	
ASD	17.0%	1.0%	<0.001, 0.26	22.0%	0.7%	<0.001, 0.26	19.0%	0.7%	<0.001, 0.26
ID	7.5%	0.8%	<0.001, 0.13	16.3%	1.0%	<0.001, 0.14	8.0%	0.8%	<0.001, 0.11
Depression	25.0%,	4.0%	<0.001, 0.19	22.1%	2.7%	<0.001, 0.19	43.8%	6.0%	<0.001, 0.22
Anxiety	26.0%	4.0%	<0.001, 0.19	21.3%	2.8%	<0.001, 0.18	46.4%	6.2%	<0.001, 0.23
CD	4.0%	0.2%	<0.001, 0.15	5.5%	0.2%	<0.001, 0.16	4.8%	0.2%	<0.001, 0.13
ODD	2.0%	0.0%	<0.001, 0.13	3.2%	0.0%	<0.001, 0.14	2.3%	0.0%	<0.001, 0.11
SUD	18.0%	4.0%	<0.001, 0.14	20.0%	4.0%	<0.001, 0.13	24.7%	4.0%	<0.001, 0.15
Non‐convicted	69.0%	89.0%	<0.001, 0.13	75.7%	84.2%	<0.001, 0.13	92.4%	99.2%	<0.001, 0.11
Violent convictions	13.0%	2.0%	<0.001, 0.13	20.0%	3.8%	<0.001, 0.14	8.6%	0.9%	<0.001, 0.11
SES level 1	42.0%	32.0%	<0.001, 0.05	42.0%	32.0%	<0.001, 0.05	42.0%	32.0%	<0.001, 0.04
SES level 2	36.0%	38.0%	<0.001, 0.05	36.0%	38.0%	<0.001, 0.05	35.0%	38.0%	<0.001, 0.04
SES level 3	22.0%	30.0%	<0.001, 0.05	22.0%	30.0%	<0.001, 0.05	23.0%	30.0%	<0.001, 0.04

*Note*: Chi Square tests for *p*‐values and Cramer's V for effect sizes.

Abbreviations: ASD, Autism spectrum disorders; CD, Conduct disorder; ID, Intellectual disability; ODD, Oppositional defiant disorder; SES, Socioeconomic Status, levels 1–3 refer to division of education level into proxy for different levels of SES; SUD, Substance use disorder.

**FIGURE 1:**
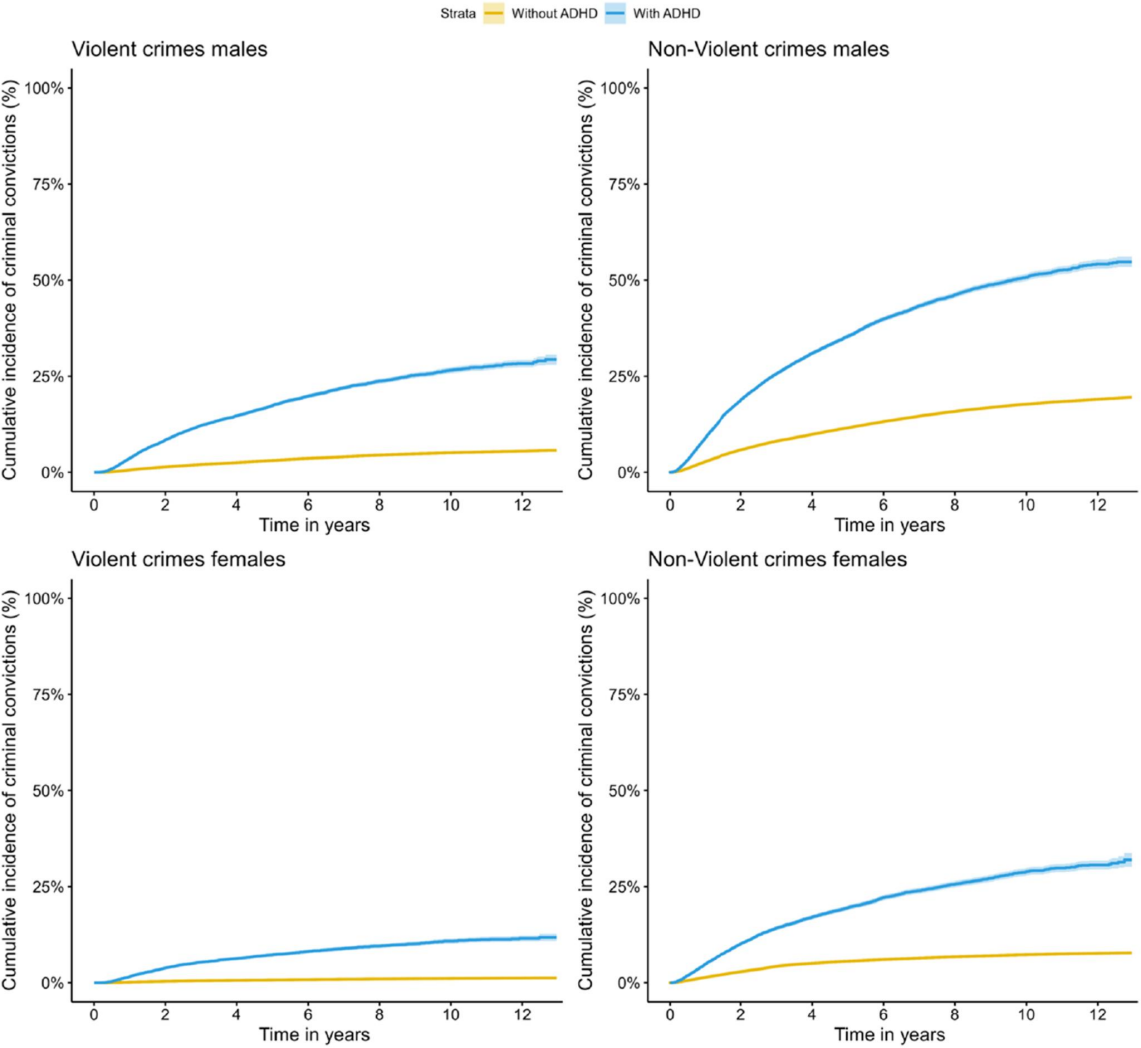
Cumulative incidence with time in years since age 15, for violent and non‐violent criminal convictions among males and females by categories with and without ADHD.

Figure 1 depicts the cumulative incidence of violent and non‐violent criminal convictions, separately for males and females. At the end of the follow‐up, the cumulative incidence of violent criminal convictions was 28.3% in males with ADHD compared to 5.5% in males without ADHD, while the corresponding cumulative incidence for non‐violent criminal convictions was 54.2% and 19.0% in males with and without ADHD. In females with ADHD, the cumulative incidence of violent and non‐violent criminal convictions was 11.5% and 30.6%, respectively, compared to 1.2% and 7.7% in females without a diagnosis of ADHD.

### Risk of being convicted of a violent or a non‐violent crime in males and females diagnosed with ADHD

Crude estimates demonstrated that males and females diagnosed with ADHD had an increased risk of violent and non‐violent criminal convictions compared to males and females without ADHD (crude HRs ranged from 3.57 to 10.50), Table 2. Test for sex differences showed that the relative risks of violent (*p* < 0.001) and non‐violent (*p* < 0.05) criminal convictions were significantly higher in females with ADHD compared to males with ADHD. Tests for differences in levels of SES showed that both males and females diagnosed with ADHD who had lower levels of SES exhibited significantly higher relative risks of both violent (*p* < 0.001) and non‐violent (*p* < 0.001) criminal convictions compared with those with a diagnosis of ADHD and higher levels of SES. Adjustments for birth year, SES, childhood, and internalizing psychiatric disorders attenuated the associations to some extent in both males and females, whilst adjustment for substance use disorders attenuated associations substantially in both sexes (HR ranged from 2.60 to 4.89), Table 2.

**TABLE 2:** Association between ADHD and violent and non‐violent criminal convictions in males and females.

Males ADHD (*n* = 4841 No ADHD (*n* = 23,369)					Males ADHD (*n* = 9670) No ADHD (*n* = 83,862)				
Violent criminal convictions					Non‐violent criminal convictions				
HR (95% CI)					HR (95% CI)				
Crude	Adjusted^a^	Adjusted^b^	Adjusted^c^	Adjusted^d^	Crude	Adjusted^a^	Adjusted^b^	Adjusted^c^	Adjusted^d^
6.03 (5.85–6.22)	5.67 (5.50–5.85)	5.17 (5.00–5.36)	4.49 (4.34–4.64)	3.74 (3.61–3.86)	3.57 (3.49–3.64)	3.45 (3.38–3.52)	3.54 (3.46–3.63)	2.98 (2.91–3.04)	2.60 (2.54–2.66)

Females ADHD (*n* = 1249) No ADHD (*n* = 4943)					Females ADHD (*n* = 3335) No ADHD (*n* = 35,074)				
Violent criminal convictions					Non‐violent criminal convictions				
HR (95% CI)					HR (95% CI)				
Crude	Adjusted^a^	Adjusted^b^	Adjusted^c^	Adjusted^d^	Crude	Adjusted^a^	Adjusted^b^	Adjusted^c^	Adjusted^d^
10.50 (9.84–11.14)	9.70 (9.11–10.33)	7.73 (7.20–8.30)	5.66 (5.27–6.09)	4.89 (4.57–5.23)	4.04 (3.90–4.18)	3.98 (3.84–4.12)	3.78 (3.64–3.93)	2.72 (2.62–2.83)	2.66 (2.56–2.76)

*Note*: Crude model.

^a^ Adjusted for birth year, and SES.

^b^ Adjusted for birth year, SES, and childhood psychiatric disorders.

^c^ Adjusted for birth year, SES, and internalizing disorders.

^d^ Adjusted for birth year, SES, and substance use disorder.

**FIGURE 2:**
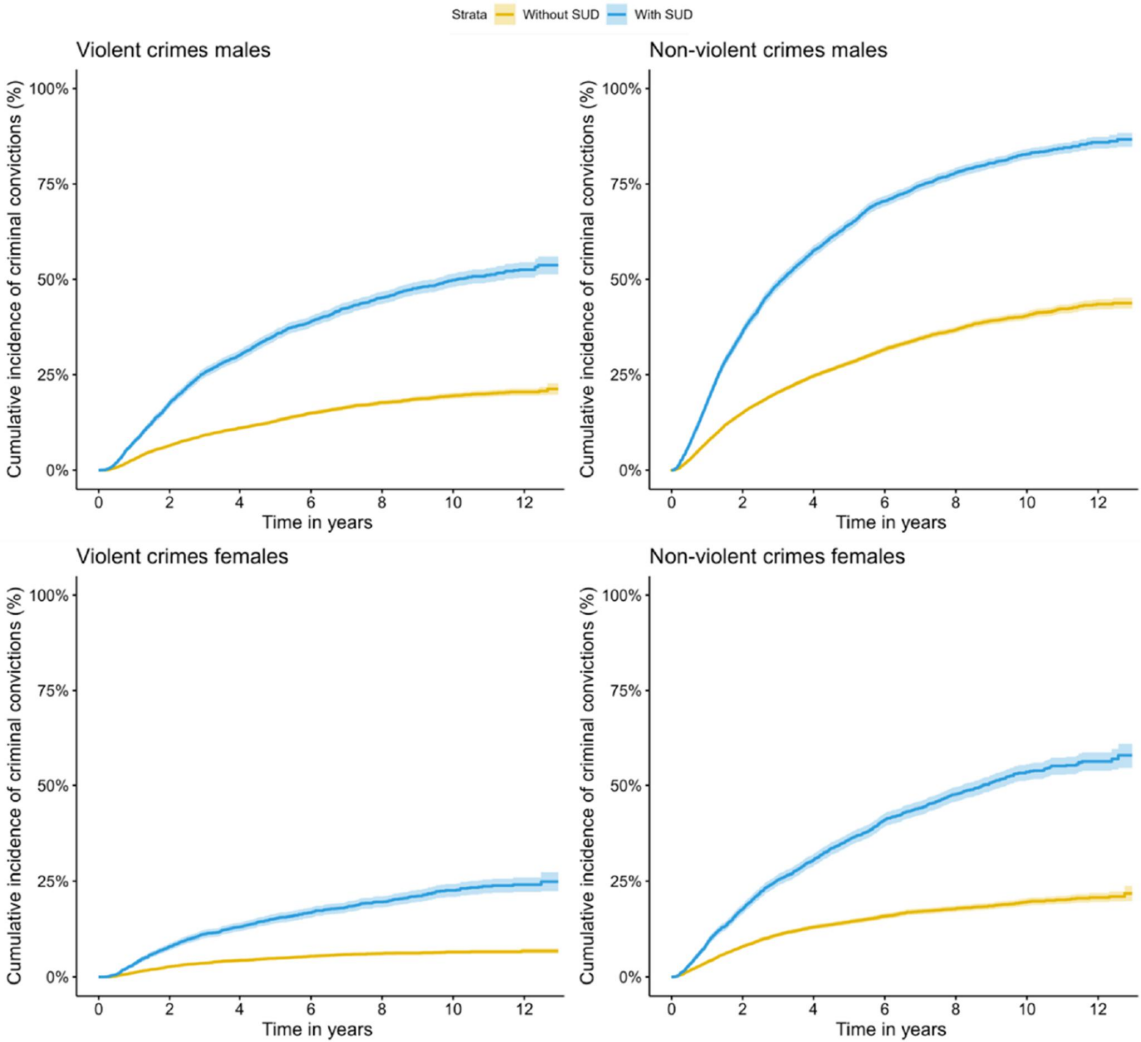
Cumulative incidence with time in years since age 15, for violent and non‐violent criminal convictions among males and females with ADHD by categories with and without SUD.

Figure 2 depicts cumulative estimates of violent and non‐violent criminal convictions in males and females with ADHD and SUD compared to males and females with ADHD without comorbid SUD. At the end of the follow‐up, the cumulative incidence of violent criminal convictions was 52.5% in males with ADHD and SUD compared to 20.5% in males with ADHD without SUD, while the corresponding cumulative incidence for non‐violent criminal convictions was 85.9% in males with ADHD and SUD and 43.5% in males with ADHD without SUD. In females with ADHD, the cumulative incidence of violent and non‐violent criminal convictions for females with ADHD and SUD was 24.1% and 56.3%, respectively, compared to 6.6% and 20.7% in females with ADHD without SUD.

### Shared familial factors

Crude estimates for the full sample showed that individuals diagnosed with ADHD had an increased risk of a violent (HR = 7.35 95% CI 7.15–7.55) and non‐violent (HR = 3.91 95% CI 3.84–4.00) criminal conviction compared to individuals without ADHD. The estimates adjusted for unmeasured familial factors attenuated compared to the crude estimates for both violent (HR = 2.64 95% CI 2.45–2.85) and non‐violent (HR = 2.12 95% CI 2.01–2.23) criminal convictions, but remained statistically significant and substantial, Table 3.

**TABLE 3:** Association between ADHD and violent and non‐violent criminal convictions adjusted for unmeasured familial factors.

	Crude	Adjusted for unmeasured familial factors
	HR (95% CI)	HR (95% CI)
Violent criminal convictions	7.35 (7.15–7.55)	3.91 (3.84–4.00)
Non‐violent criminal convictions	2.64 (2.45–2.85)	2.12 (2.01–2.23)

### Sensitivity analyses

The sensitivity analysis where ADHD could be present at any time (before or after the criminal conviction) remained robust, see Table S1. The sensitivity analysis where those who received pharmacological treatment for ADHD were excluded indicated increased estimates across all groups (for example crude HR = 11.02 for violent criminal convictions in males compared to HR = 6.03 in the corresponding main analysis), Table S2.

## DISCUSSION

In this population‐based study, we demonstrated that ADHD is a strong risk factor for violent and non‐violent criminal convictions in both males and females, although the magnitudes of the association were different between males and females, especially for violent criminal convictions. Notably, we found that individuals with ADHD who had lower levels of SES exhibited significantly higher relative risks of both violent and non‐violent criminal convictions compared to individuals with ADHD and higher levels of SES. This finding underscores the complexity of the relationship between ADHD and criminal outcomes. Furthermore, our study found ADHD to be an independent risk factor for criminal convictions after adjusting for comorbid psychiatric disorders and unmeasured familial factors. This highlights the need for future research to further identify modifiable mediating factors (i.e., on the causal pathway from ADHD to criminal convictions) that could be targeted for intervention. Among the comorbid psychiatric disorders, SUD explained most of the risk of criminal convictions associated with having ADHD. SUD was also associated with a higher absolute risk of both violent and non‐violent criminal convictions in males and females with ADHD compared to males and females with ADHD without comorbid SUD.

### Sex differences in ADHD and criminal convictions

As expected and consistent with previous research, we found that both ADHD (Arnold, 1996; Gaub & Carlson, 1997; Mowlem et al., 2019; Nøvik et al., 2006; Ramtekkar et al., 2010; Rucklidge, 2010; Willcutt, 2012) and criminal convictions (Gottfredson & Hirschi, 1990; Staniloiu & Markowitsch, 2012) were more common in males than in females. Our results also demonstrated higher relative risks for violent and non‐violent criminal convictions in females with ADHD compared to males with ADHD. In contrast to the higher relative risk in females, we found a substantially higher absolute risk of violent (28.3% compared to 11.5%) and non‐violent (54.2% compared to 30.6%) criminal convictions among males with ADHD compared to females with ADHD. Our sex‐specific results are in line with a previous Danish register‐based study (Mohr‐Jensen et al., 2019), but in contrast with a previous register‐based study from Australia (Silva et al., 2014) which did not find a higher relative risk of criminal conviction in females with ADHD compared to males with ADHD. Thus, research from other geographical regions is needed to explore the generalizability of the observed sex differences underlying the association between ADHD and criminal convictions. We note that the association between ADHD and criminal convictions in females was higher in our study (HR = 4.28 95% CI 3.99–4.60) compared to a previous Danish register‐based study (HR = 2.2 95% CI 1.5–3.1) (Mohr‐Jensen et al., 2019). One possible explanation for this could be the larger number of females in our study (37% of the ADHD sample compared to 15%). The identified differences in risk between males and females (with higher absolute risk in males and higher relative risk in females) have important implications. Sex needs to be considered carefully in future prediction modeling research (Senior et al., 2021) to develop effective risk assessment instruments as well as effective treatment suggestions for both males and females with ADHD. The higher relative risk of criminal convictions in females with ADHD may suggest a more severe phenotype of ADHD in females. For example, females with ADHD may have to present more severe symptoms than males to receive a diagnosis of ADHD (Mohr‐Jensen et al., 2019). However, this needs to be further examined, for example, with future studies using less male dominant samples. The higher relative risk in females with ADHD could also be related to the later diagnosis of females with ADHD compared to males, which, in turn, could delay interventions and treatment, and therefore lead to more negative consequences (Mohr‐Jensen et al., 2019).

### Socio‐economic status and criminal convictions in ADHD

In consistency with prior research findings (Mohr‐Jensen et al., 2019), our study underscores the substantial association between SES and criminal convictions within individuals diagnosed with ADHD. Specifically, we found that lower levels of SES in individuals with ADHD were linked to higher relative risks of both violent and non‐violent criminal convictions compared to individuals diagnosed with ADHD who had higher levels of SES. While several potential explanations exist for this observed association, encompassing factors such as limited educational opportunities, unemployment, restricted access to mental health services, and social support deficiencies (Savolainen et al., 2018), it is worth noting that unmeasured familial factors have also been proposed as contributors to this association (Sariaslan et al., 2014; Sariaslan et al., 2021).

Regardless of the specific mechanisms underpinning the relationship between lower SES and a heightened risk of criminal convictions in the context of ADHD, it is imperative that we continue to investigate this complex interplay. Such exploration is vital not only for gaining deeper insights into the contributing factors but also for shaping evidence‐based interventions and policies aimed at reducing disparities and offering support to individuals with ADHD, particularly those from disadvantaged socioeconomic backgrounds.

### Comorbid psychiatric disorders

Our findings suggest that childhood psychiatric disorders and internalizing disorders explained part of the risk of criminal convictions in individuals with ADHD, but to a lesser extent than SUD. SUD also further increased the absolute risk of violent and non‐violent criminal convictions compared to ADHD without SUD in both males and females. This finding suggests that SUD plays an important role in the risk of violent and non‐violent criminal convictions in individuals with ADHD and is in line with those of previous research (Dalsgaard et al., 2013; Mohr‐Jensen et al., 2019; Mohr‐Jensen & Steinhausen, 2016; Retz et al., 2021). These findings indicate that future prediction modeling research focusing on the risk of criminal convictions in ADHD should consider including detailed information about SUD, symptoms, traits, and treatments.

### Unmeasured familial factors

Several studies have shown that ADHD (Chen et al., 2017; Faraone & Larsson, 2019), criminality (Frisell et al., 2011), and psychiatric disorders (Polderman et al., 2015) aggregate in families, and one previous study has shown that familial factors contribute to the association between ADHD and criminal convictions (Lundström et al., 2014). In line with this, we found that unmeasured familial factors partly explained the association between ADHD and the risk of criminal convictions. Previous research indicate that a lack of impulse control is an important mechanism behind the association between ADHD and criminal convictions (Young & Thome, 2011) and that impulsivity is highly heritable (Bevilacqua & Goldman, 2013). This heritability in combination with adverse environmental factors (e.g., related to upbringing (Frisell et al., 2011)) could be one, among many, underlying mechanisms explaining the association between ADHD and criminal convictions. Lack of impulse control could thereby be an important target for intervention.

### Pharmacological treatment

Sensitivity analyses excluding participants that received ADHD medication resulted in higher risks for criminal convictions compared to analyses including all cases of ADHD whether they received pharmacological treatment or not. These results indicate that ADHD medication may reduce the risk of both violent and non‐violent criminal convictions in males and females with ADHD. These results are in line with those of previous studies and further strengthen the role of pharmacological treatment as an important part of ADHD treatment (Lichtenstein et al., 2012; Mohr‐Jensen et al., 2019).

### Strengths and limitations

In our current study, we relied on national population register data, using clinical diagnoses to assess psychiatric conditions and criminal convictions to assess the outcome. Register‐based research is characterized by a notable strength: the occurrence of missing data is typically minimal or nonexistent. In our specific sample, after exclusions, we encountered 111 cases with missing data on parental education/SES, while all other covariates had complete data. Nevertheless, it is important to acknowledge a potential limitation associated with our method of using clinical diagnoses and ADHD medication prescriptions as a measure of ADHD. This approach may not fully capture individuals with milder ADHD symptoms or those who do not seek healthcare for various reasons, thus leading to an underrepresentation of ADHD in our study. However, it is worth noting that the observed prevalence of clinically diagnosed ADHD in our sample (3.5%) aligns closely with findings from previous studies (Fayyad et al., 2017; Jangmo et al., 2021; Polanczyk et al., 2014).

Another potential concern when using register data is exposure misclassification, particularly false negatives. This means that individuals with ADHD symptoms who have not received an official diagnosis may be mistakenly categorized as unexposed. If this misclassification is nondifferential, it could potentially weaken the estimated associations. To address this limitation, we conducted sensitivity analyses allowing for the presence of ADHD at any point (before or after criminal convictions), which showed somewhat attenuated estimates while maintaining robust associations (see Table S1). Additionally, misclassification in other variables, such as comorbid psychiatric and substance use disorders, could also introduce bias into our findings. We used a time‐fixed approach to examine comorbid psychiatric disorders, but this method lacks information on the timing of diagnoses in relation to ADHD and criminal convictions. Future research should explore the timing of psychiatric comorbidities in greater detail, employing time‐sensitive approaches.

One significant limitation to consider is that our data only extend up to 2013. This is due to the unavailability of more recent data from Swedish national registers, which restricts our ability to assess potential changes or trends beyond that timeframe. Nevertheless, we anticipate that the associations we observed are likely to remain similar, but this assumption needs to be examined using newer data.

Due to the limited number of females with ADHD who had criminal convictions, we were unable to perform separate analyses for males and females in the analyses including unmeasured familial factors. Instead, we adjusted for sex in our analyses that included both genders to account for potential gender differences. However, it is important to recognize that the lack of statistical power in this analysis is a limitation.

Our approach to assessing the impact of pharmacological treatment has limitations. Nevertheless, previous research, including our own studies (Lichtenstein et al., 2012), has employed advanced pharmaco‐epidemiological methods (within‐individual approaches) to investigate the specific link between pharmacological treatment in individuals with ADHD and criminality. These studies consistently indicate lower rates of criminal conviction during periods of pharmacological treatment compared to non‐treatment periods among individuals with ADHD (Lichtenstein et al., 2012), aligning with our own findings. One related limitation of our study is that we were unable to account for other forms of ADHD treatment.

Our study revealed higher rates of criminal convictions among individuals with ADHD compared to those without, with non‐violent convictions outnumbering violent ones. This trend mirrors findings from previous register‐based studies (Mohr‐Jensen et al., 2019; Silva et al., 2014). For instance, in our study, 31% of both males and females with ADHD had criminal convictions, while the corresponding rate for individuals without ADHD was 11%. These rates are consistent with those reported by The Swedish National Council for Crime Prevention for Sweden (Brottsförebyggande rådet; Brå, 2022), and criminal conviction rates across Europe are also similar (Cepeda, 2016; Dolmén, 2001). However, comparing our results to those in the United States may be challenging due to significant differences in legal and criminal justice systems across countries (Farrington et al., 2004).

### Conclusions

ADHD is a strong risk factor for both violent and non‐violent criminal convictions in males and females even after adjustments for psychiatric comorbidities and unmeasured familial factors. ADHD posed a higher relative risk of criminal convictions in females and a higher absolute risk in males, indicating that sex differences need to be considered carefully in future research. Individuals with ADHD from lower socioeconomic backgrounds faced a higher risk of criminal convictions. This SES disparity underscores the multifaceted nature of ADHD's impact on criminal outcomes and highlights the importance of considering socioeconomic disparities in future research. Childhood psychiatric disorders and internalizing disorders influenced the association between ADHD and criminal convictions to some extent. SUD explained a substantial part of the unadjusted association between ADHD and criminal convictions and further increased the absolute risk of criminal convictions compared to ADHD without SUD in both males and females. Unmeasured familial confounding explained part of, but not fully, the elevated risk of criminal convictions in males and females with ADHD. Considering the increased rate of criminal convictions in individuals with a diagnosis of ADHD and especially the high relative risks in females with ADHD future research should further examine this in less male dominant samples. Individuals with ADHD and comorbid SUD should be targeted for treatment and prevention considering the elevated risk for criminal convictions.

## AUTHOR CONTRIBUTIONS

**Anna‐Karin Ångström**: Conceptualization; data curation; formal analysis; investigation; methodology; project administration; resources; software; validation; visualization; writing—original draft; writing—review and editing. **Anneli Andersson**: Conceptualization; data curation; formal analysis; methodology; software; supervision; validation; writing—original draft; writing—review and editing. **Miguel Garcia‐Argibay**: Data curation; formal analysis; methodology; software; validation; writing—review and editing. **Zheng Chang**: Conceptualization; validation; writing—review and editing. **Paul Lichtenstein**: Conceptualization; methodology; validation; writing—review and editing. **Brian M. D'Onofrio**: Conceptualization; methodology; validation; writing—review and editing. **Catherine Tuvblad**: Conceptualization; methodology; supervision; validation; writing—review and editing. **Laura Ghirardi**: Conceptualization; methodology; software; supervision; validation; visualization; writing—review and editing. **Henrik Larsson**: Conceptualization; investigation; methodology; project administration; resources; supervision; validation; visualization; writing—review and editing.

## CONFLICT OF INTEREST STATEMENT

Henrik Larsson reports receiving grants from Shire Pharmaceuticals; personal fees from and serving as a speaker for Medice, Shire/Takeda Pharmaceuticals and Evolan Pharma AB; and sponsorship for a conference on attention‐deficit/hyperactivity disorder from Shire/Takeda Pharmaceuticals and Evolan Pharma AB, all outside the submitted work. Henrik Larsson is Editor‐in‐Chief of JCPP Advances.

## ETHICAL CONSIDERATIONS

The study had ethical approval from the Regional Ethical Review Board in Stockholm, Sweden (Dnr 2013/862–31/5). The requirement for informed consent was waived because the study was register‐based and data on the included individuals were de‐identified. The investigation conforms to the 1964 Helsinki declaration and its later amendments or comparable ethical standards.

## Supporting information

Supporting Information S1

## Data Availability

Data may be obtained from a third party and are not publicly available. The Public Access to Information and Secrecy Act in Sweden prohibits individual‐level data to be publicly available. Researchers who are interested in replicating this study can apply for individual level data at Statistics Sweden: www.scb.se/en/services/guidance‐for‐researchers‐and‐universities/.
