# Supplementary material for: Criminal convictions in males and females diagnosed with attention deficit hyperactivity disorder: A Swedish national registry study
Source: JCPP Adv. 2024 Jan 20;4(1):e12217. doi: 10.1002/jcv2.12217 (PMC10933617; doi:10.1002/jcv2.12217)
Supplement: Supplementary file 1 — Supporting Information S1 [file JCV2-4-e12217-s001.docx]

| **Males ADHD (n = 5,381)** | | | | **No ADHD (n=** **22,829)** | | | **Males ADHD (n = 10,577)** | | | | **No ADHD (n=** **82,955)** | | |
| --- | --- | --- | --- | --- | --- | --- | --- | --- | --- | --- | --- | --- | --- |
| **Violent Criminal Convictions** | | | | | | | **Non-Violent Criminal Convictions** | | | | | | |
| **HR (95% CI)** | | | | | | | **HR (95% CI)** | | | | | | |
| Crude | Adjusted^a^ | Adjusted^b^ | | | Adjusted^c^ | Adjusted^d^ | Crude | Adjusted^a^ | Adjusted^b^ | | | Adjusted^c^ | Adjusted^d^ |
| 5.54  (5.38-5.71) | 5.15 (5.00-5.31) | 4.82 (4.66-4.98) | | | 4.13  (4.00-4.27) | 3.48  (3.37-3.59) | 3.15  (3.09-3.21) | 3.02 (2.96-3.08) | 3.15  (3.08-3.22) | | | 2.61  (2.56-2.67) | 2.31  (2.26-2.36) |
| **Females ADHD (n = 1,378)** | | | **No ADHD (n =** **4,814)** | | | | **Females ADHD (n = 3,754)** | | | **No ADHD (n =** **34,655)** | | | |
| **Violent Criminal Convictions** | | | | | | | **Non-Violent Criminal Convictions** | | | | | | |
| **HR (95% CI)** | | | | | | | **HR (95% CI)** | | | | | | |
| Crude | Adjusted^a^ | Adjusted^b^ | | | Adjusted^c^ | Adjusted^d^ | Crude | Adjusted^a^ | Adjusted^b^ | | | Adjusted^c^ | Adjusted^d^ |
| 9.76  (9.20-10.37) | 8.97  (8.44-9.52) | 7.39  (6.90-7.91) | | | 5.38  (5.01-5.77) | 4.68  (4.38-4.99) | 3.79  (3.66-3.92) | 3.71  (3.59-3.84) | 3.60  (3.47-3.73) | | | 2.56  (2.47-2.66) | 2.53  (2.44-2.62) |

**Table S1. Association Between ADHD and Convictions of Violent and Non-Violent Crimes in Males and Females. Sensitivity analysis: ADHD Can be Present at any Time.**

*Note.* Crude model; Adjusted^a^ = adjusted for birth year and SES; Adjusted^b^ = adjusted for birth year, SES and childhood psychiatric disorders, Adjusted^c^ = adjusted for birth year, SES and internalizing disorders, Adjusted^d^ = adjusted for birth year, SES and substance use disorder.

**Table S2. Association Between Individuals with ADHD not Receiving Pharmacological Treatment and Convictions of Violent and Non-Violent Crimes in Males and Females.**

| **Males (n=2,356)** | | | | | | **Males (n=5,009)** | | | | | |
| --- | --- | --- | --- | --- | --- | --- | --- | --- | --- | --- | --- |
| **Violent Criminal Convictions** | | | | | | **Non-Violent Criminal Convictions** | | | | | |
| **HR (95% CI)** | | | | | | **HR (95% CI)** | | | | | |
| Crude | Adjusted^a^ | Adjusted^b^ | | Adjusted^c^ | Adjusted^d^ | Crude | Adjusted^a^ | Adjusted^b^ | Adjusted^c^ | | Adjusted^d^ |
| 11.02  (10.00-12.015) | 8.88 (8.05-9.80) | 6.08  (5.47-6.76) | | 6.84  (6.20-7.55) | 5.33 (4.83-5.88) | 9.36  (8.78-9.97) | 8.05 (7.56-8.59) | 7.42  (6.94-7.93) | 6.85  (6.42-7.30) | | 5.39  (5.05-5.75) |
| **Females (n=498)** | | |  | | | **Females (n=1,201)** | | | |  | |
| **Violent Criminal Convictions** | | | | | | **Non-Violent Criminal Convictions** | | | | | |
| **HR (95% CI)** | | | | | | **HR (95% CI)** | | | | | |
| Crude | Adjusted^a^ | Adjusted^b^ | | Adjusted^c^ | Adjusted^d^ | Crude | Adjusted^a^ | Adjusted^b^ | Adjusted^c^ | | Adjusted^d^ |
| 37.62  (29.67-47.71) | 30.58 (24.11-38.80) | 13.66  (10.59-17.62) | | 15.12 (11.88-19.23) | 10.36  (8.14-13.17) | 18.84  (16.39-21.66) | 16.86  (14.67-19.38) | 13.47  (11.65-15.58) | 10.85  (9.43-12.49) | | 8.68  (7.54-9.99) |

*Note.* Crude model; Adjusted^a^ = adjusted for birth year, and SES; Adjusted^b^ = adjusted for birth year, SES, and childhood psychiatric disorders, Adjusted^c^ = adjusted for birth year, SES, and internalizing disorders, Adjusted^d^ = adjusted for birth year, SES, and substance use disorder.

**Supplementary Table 1.** **Prevalence of ADHD, Psychiatric Comorbidities, Criminal Convictions and SES in the Excluded Study Population.**

|  | **Total (n=305,111)** | | **Males (n = 169,027)** | | **Females (n =136,084)** | |
| --- | --- | --- | --- | --- | --- | --- |
|  | **ADHD**  **(n=7,990)** | **Non-ADHD**  **(n= 297121)** | **ADHD  (n =6,008)** | **Non-ADHD  (n = 163,019)** | **ADHD  (n =1982)** | **Non-ADHD  (n =134,102)** |
| **ASD** | 14.0% | 0.4% | 14.0% | 0.5% | 17.0% | 0.3% |
| **ID** | 12.0% | 0.7% | 12.0% | 0.8% | 11.0 % | 0.6% |
| **Depression** | 23.0% | 3.0% | 21.0% | 3.0% | 32.0% | 3.0% |
| **Anxiety** | 26.0% | 3.0% | 23.0% | 3.0% | 35.0% | 3.1% |
| **CD** | 9.0% | 0.2% | 10.0% | 0.4% | 8.0% | 0.0% |
| **ODD** | 2.0% | 0.0% | 2.0% | 0.0% | 2.3% | 0.0% |
| **SUD** | 36.0% | 5.0% | 37.0% | 7.0% | 34.0% | 2.0% |
| **Non-Convicted** | 35.0% | 81.0% | 28.0% | 70.0% | 58.0% | 93.0% |
| **Violent Convictions** | 46.0% | 9.0% | 55.0% | 16.0% | 20.0% | 1.0% |
| **SES level 1** | 39.0% | 37.0% | 40.0% | 38.0% | 35.0% | 35.0% |
| **SES level 2** | 34.0% | 31.0% | 34.0% | 32.0% | 32.0% | 31.0% |
| **SES level 3** | 27.0% | 32.0% | 26.0% | 30.0% | 33.0% | 34.0% |

*Note.* ASD= Autism spectrum disorders; ID= Intellectual disability; CD= Conduct disorder; ODD= Oppositional defiant disorder; SUD= Substance use disorder;

SES = Socioeconomic Status, levels 1-3 refer to division of education level into proxy for different levels of SES.

**Supplementary Table 2. Average age of ADHD-diagnosis and First Criminal Conviction in the Study Population.**

|  | **Full Sample** | | **Males** | | **Females** | |
| --- | --- | --- | --- | --- | --- | --- |
|  | **ADHD** | **No**  **ADHD** | **ADHD** | **No ADHD** | **ADHD** | **No ADHD** |
|  | **Age M (SD)** | | **Age M (SD)** | | **Age M (SD)** | |
| **ADHD-diagnosis** | 16.00 (4.47) | - | 15.06 (4.51) | - | 17.01 (4.09) | - |
| **Any Criminal Conviction** | 17.02 (2.18) | 18.00  (2.50) | 16.97 (2.15) | 17.71 (2.56) | 17.17 (2.27) | 17.23 (2.30) |
| **Violent Criminal Conviction** | 20.10 (3.30) | 21.50 (3.34) | 19.78 (3.22) | 21.44 (3.35) | 20.56 (3.27) | 21.53 (3.32) |
| **Non-Violent Criminal Conviction** | 19.36 (3.23) | 21.10 (3.50) | 19.01 (3.14) | 21.00 (3.50) | 19.94 (3.30) | 21.25 (3.42) |

**Supplementary Table 3. ICD-codes.**

| **Disorder** | **ICD-9** | **ICD-10** |
| --- | --- | --- |
| **ADHD** | 314 | F90 |
| **Autism spectrum disorders** | 299 | F84.0-5, F84.8, F84.9 |
| **Intellectual disability** | 317-319 | F70-F79 |
| **Depression** | 296B, 298A, 296W, 311, 300E, 300F, 309A, 309B | F32, F33, F34, 381, F488, 432 |
| **Anxiety** | 300A, 300C, 300D | F40, F41, F42 |
| **Conduct disorder** | 312.89 | F91.9 |
| **Oppositional defiant disorder** | 313.81 | F91.3 |
| **Substance use disorder** | 291, 292, 303, 304, 305 | F10, F11, F12, F13, F14, F15, F16, F17, F18, F19 |

*Note.* ICD-codes refer to Swedish versions.
